# Electronic Structure of Polyethylene: Role of Chemical, Morphological and Interfacial Complexity

**DOI:** 10.1038/s41598-017-06357-y

**Published:** 2017-07-21

**Authors:** Lihua Chen, Tran Doan Huan, Rampi Ramprasad

**Affiliations:** Department of Materials Science & Engineering and Institute of Materials Science, University of Connecticut, 97 North Eagleville Rd., Unit 3136, Storrs, CT 06269-3136 USA

## Abstract

The electronic structure of an insulator encodes essential signatures of its short-term electrical performance and long-term reliability. A critical long-standing challenge though is that key features of the electronic structure of an insulator (and its evolution) under realistic conditions have not been entirely accessible, either via experimental or computational approaches, due to the inherent complexities involved. In this comprehensive study, we reveal the role of chemical and morphological imperfections that inevitably exist within the technologically important prototypical and pervasive insulator, polyethylene (PE), and at electrode/PE interfaces. Large-scale density functional theory computations and long-time molecular dynamics simulations were employed to accurately recover, explain and unravel a wide variety of experimental data obtained during the electrical degradation of PE. This scheme has allowed us to directly and realistically address the role of chemical, morphological and interfacial complexity in determining electronic structure. These efforts take us a step closer to understanding and potentially controlling dielectric degradation and breakdown.

## Introduction

Polymers are widely used in electric and electronic devices, e.g., capacitors^1–8^, transistors^9, 10^, fuel cell membranes^11, 12^ and high-voltage cables^13, 14^. The insulating behavior of polymers—or any material for that matter—becomes progressively (and in many cases, irreversibly) degraded over time, especially when they are exposed to heat, light, oxygen, moisture, mechanical stress, and the high electric fields encountered during operation^14–17^. This process ultimately leads to dielectric breakdown, the event by which the material sharply loses its insulating characteristics. Typically, polymer degradation involves a wide variety of physical and chemical processes, spanning over several length and time scales. The highly complicated and coupled nature of these processes render detailed mechanistic studies far from being tractable, both computationally^18–24^ and experimentally^14, 17, 24–28^, despite extensive recent efforts aimed at the rational design of polymer dielectrics^3, 6, 8, 29–32^.

One characteristic aspect of a material that encodes details of its insulating behavior is its electronic structure^14, 28^. Although perfect, defect-free, single-crystalline “good” insulators may have majestic band gaps of over 8 eV, various types and classes of imperfections erode the electronic structure. Real materials, especially polymers, are never single-crystals, nor are they devoid of chemical imperfections in bulk or close to interfaces with other materials (e.g., electrode metals). Such imperfections translate to features in the electronic structure, such as defect or “trap” states within the band gap, alteration of the band edge positions leading to a decrease of the band gap value, and undesirable degrees of offsets between band edges across interfaces (e.g., between the insulator and an electrode)^14, 20, 33^. These factors control both charge transport within the insulator and charge injection into the insulator (from electrodes)^14, 33^. Even if the insulating material was perfect to begin with, extrinsic factors, such as heat, light, electric fields, etc., will gradually introduce imperfections in the material, thus dynamically degrading its electronic structure, and consequently, leading to electron avalanches and dielectric breakdown^14, 17^.

**Figure 1:**
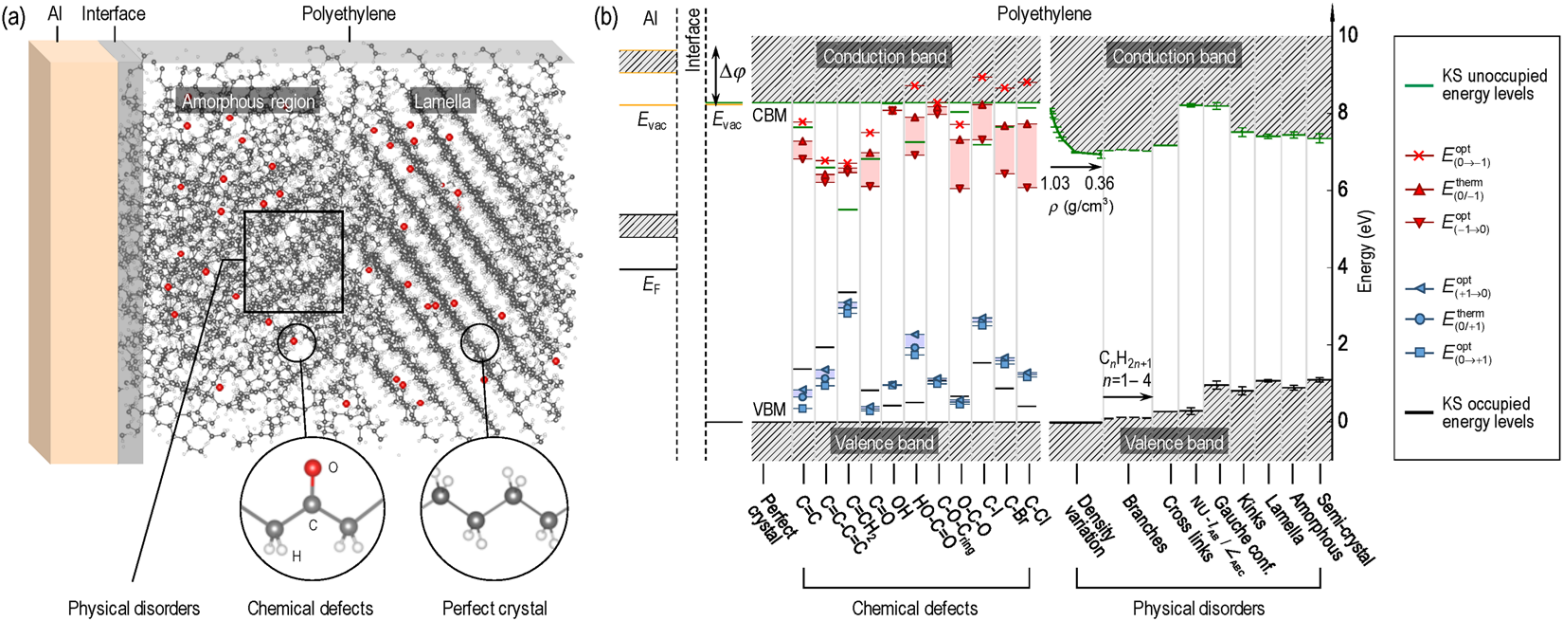
(**a**) Schematic structure of an Al/PE interface, containing crystalline and amorphous regions, and populations of chemical defects. Here, C, H, and O atoms are shown in black, white, and red, respectively. (**b**) Electronic structure of Al/PE interfaces and bulk PE with imperfections. *E*_vac_, Δ*φ*, *E*_F_, CBM and VBM are the vacuum level, the vacuum energy shift, the Fermi level of Al, and the conduction band minimum and the valence band maximum of PE, respectively. Error bars of VBM and CBM are obtained by determining the standard deviations from 10 different configurations considered for each disorder/defect. Shaded region of *E*_vac_ is induced by the O-containing groups at Al/PE interfaces. ${E}_{\mathrm{(0/}\pm \mathrm{1)}}^{{\rm{therm}}}$ and ${E}_{\mathrm{(0}\leftrightarrows \pm \mathrm{1)}}^{{\rm{opt}}}$ are the thermodynamic and optical charge transition levels. All energy levels are with respect to the average C-1*s* core level of the perfect crystal PE whose VBM is set to 0 eV.

In the present work, we focus on polyethylene (PE), which is one of the most widely used and studied polymeric insulators^20, 24, 26–28, 33–46^. A plethora of careful experimental studies, e.g., X-ray diffraction and infrared spectrum (IR), are available that have probed its physical, chemical and electronic structures, as a function of environmental factors that lead to degradation products in this material^24, 41–46^. Despite these past efforts, and despite parallel computational efforts, a clear understanding of the electronic structure of “realistic” PE, is still not at hand. A snapshot of such a “realistic” situation for the case of PE interfacing with a metallic electrode (in this case Al) is portrayed in Fig. 1(a), whose complexity makes it immediately obvious why this problem continues to be challenging. In short, bulk PE, as any other polyolefins, is a mixture of amorphous and crystalline (within lamellas) regions^41^. Moreover, evidences from IR spectra have shown that a variety of (point-like) physical disorders, e.g., kinks (with bands in a range of 1,300 – 1,400 cm^−1^)^42^ and branches (methyl groups with a band at 2,962 cm^−1^)^41^, and chemical defects such as carbonyl (C=O with a band at 1,720 cm^−1^)^43–45^, are present in PE. This complicated “blend”, which is created as-prepared, progressively evolves during or post operation. Computations have not been easy to perform at the requisite level of theory for such large systems, and so are typically undertaken for parts of (idealized versions of) the real system^33, 35, 38, 39^. Available luminescence and charge injection barrier measurements are hard to unravel due to the multitude of physical and chemical complexities involved, and are hard to correlate to available computational work^37^.

This contribution attempts to fill the above gap by charting *comprehensively* the electronic structure of realistic PE, inclusive of a majority of its chemo-physical complexity at one consistent (and high) level of theory using state-of-the-art large scale density functional theory (DFT)^47, 48^ calculations and molecular dynamics simulations. A variety of chemical, physical, interfacial and morphological imperfections and disorders (requiring enormous unit cells containing up to 2,400 atoms) have been explicitly considered, and their direct role in manipulating the electronic structure of PE has been revealed. Figure 1(b) shows a summary of our main findings in one unified portrayal, created using one common energy reference. The effect of the interface with Al (containing varying amounts of the inevitable O) on the charge injection barriers, the trap states due to a variety of chemical defects within the band gap, and modulation of the band edge positions due a plethora of physical and morphological imperfections can all be clearly seen. In addition to revealing the complex electronic structure of realistic PE, which, in and of itself, is a key major and useful outcome of this work, the results provide a basis for understanding existing experimental results. The electronic structure picture also may be a starting point for building phenomenological transport models in which the populations of various defects may be treated as variables and tracked alongside experimental measurements. Overall, it is hoped that this work will spur further studies leading to a better understanding of key factors that control dielectric degradation and breakdown.

## Results

### Electronic structure of polyethylene

**Figure 2:**
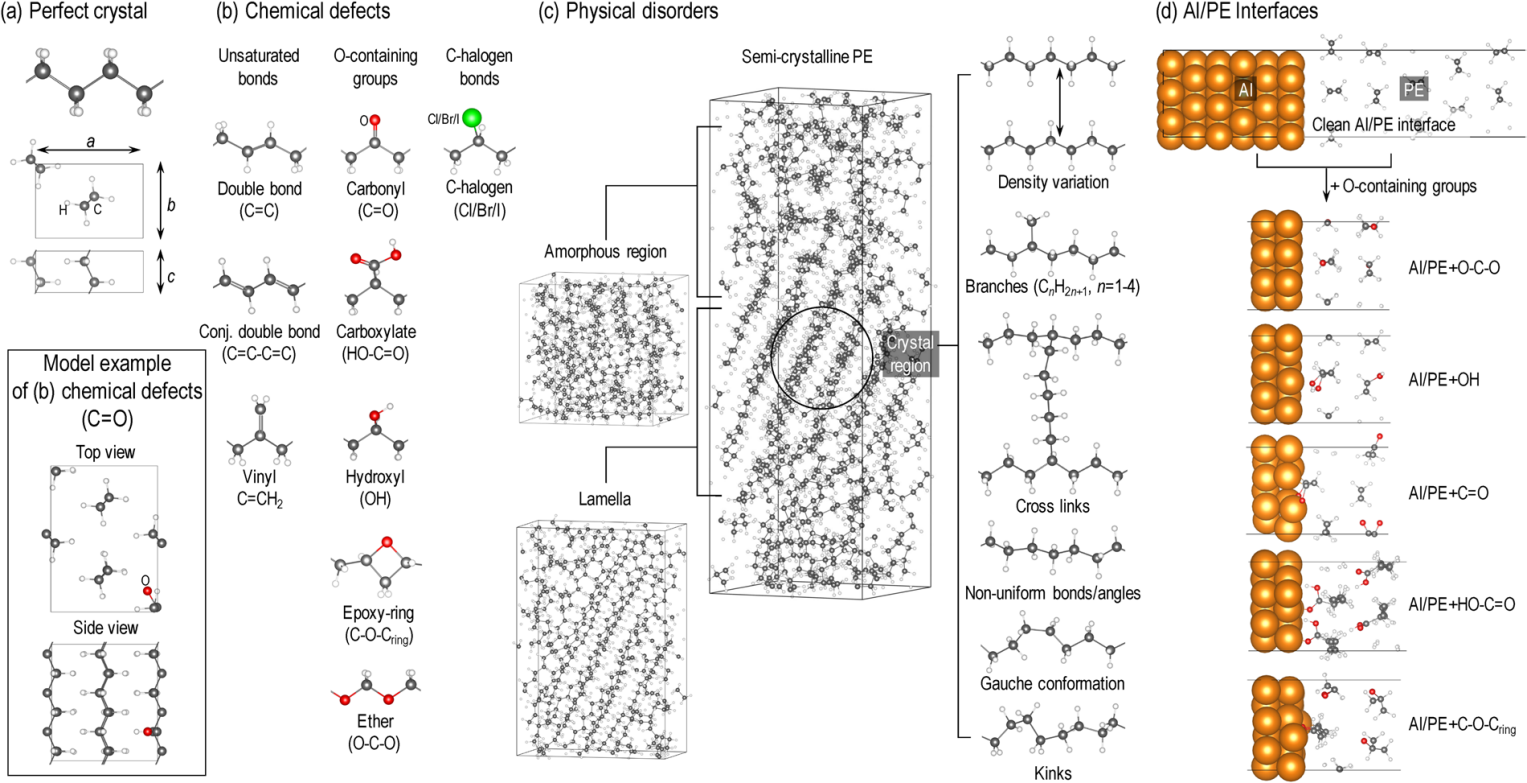
Sketched structures of the perfect crystalline PE (**a**), PE chemical defects (**b**), PE physical disorders (**c**), and Al/PE interfaces (**d**) considered in this work. Al, C, H and O atoms are shown in gold, black, white, and red, respectively.

Crystalline PE, as depicted in Fig. 2(a), has two all-trans CH_2_ chains packed in an orthorhombic unit cell with measured lattice parameters *a* = 7.12 Å, *b* = 4.85 Å, and *c* = 2.55 Å^41^. These chains, characterized by strong intra-chain hybridized *sp* ^3^ bonds, are held together by rather weak van der Waals (vdW) interactions. The computed band gap of the crystalline PE is 8.28 eV, agreeing well with the measured value of 8.8 eV. Its computed electronic structure, shown in Fig. S1 of Supplementary Information (SI), reveals that the valence band maximum (VBM) of PE is located at the G and S reciprocal space points, dominated by the intra-chain *sp* ^3^-*σ* bonds. The conduction band minimum (CBM) is located between the G and S points, characterized by hybridized anti-bonding orbitals between adjacent PE chains. Thus, modifications of the chains and/or the distance between them may alter significantly the electronic properties of PE.

### Polyethylene with defects/disorders

Imperfections in PE may be of physical or chemical nature. The former involves conformational or density deviations from the perfect crystalline PE structure^41, 42^ while the latter includes unsaturated bonds, impurities, and possible reaction products^43–46^. In this work, the defects/disorders were constructed and studied, unveiling their possible roles in manipulating the electronic and carrier transport properties of PE. Such studies are non-trivial, as high-fidelity modeling of polymers in the required large scales (to encompass physical disorders) is intrinsically challenging^3^.

#### Chemical defects

From the IR-based experimental works^43–45^, it is known that unsaturated bonds, e.g., double bond C=C, conjugate double bond C=C–C=C, and vinyl C=CH_2_, and oxygen-containing groups, e.g., carbonyl C=O, carboxylate HO–C=O, hydroxyl OH, ether O–C–O, and epoxy-ring C–O–C_ring_, are common chemical defects in PE. For halogen doped PE films, additional C-halogen (Cl, Br and I) bonds inevitably exist^46, 49^. All of these defects, graphically shown in Fig. 2(b), were considered in this work. The concentrations of these defects were chosen in the model systems to be approximately 3.24 mol/L (1 defect in a 1 × 2 × 3 supercell of PE). Although this value is greater than the experimental counterpart ($\mathop{ < }\limits_{ \tilde {}}$ 1 mol/L)^43–45^, the separation between defects is already about 7 Å and above, being suitably large to model the experimentally used density of defects. The electronic structure of PE with these defects were examined by computing the Kohn-Sham energy levels, and thermodynamic and optical charge transition levels involving different charged states. These characteristic signatures can be related to measured luminescences spectra, as done previously^3, 26, 37^.

Figure 1(b) shows that additional occupied and unoccupied energy levels were introduced within the PE band gap. For unsaturated bonds, e.g., C=C, the electron (hole) trapping levels are ascribed to the *π* bonding (${\pi }^{\ast }$ anti-bonding), for which the related *p*_*z*_ orbitals do not align, reducing the overlaps. Among these defects, the electron and hole trap depths, i.e., ${E}_{{\rm{t}}}^{{\rm{e}}}$ and ${E}_{{\rm{t}}}^{{\rm{h}}}$, defined as the shifts of the CBM or VBM induced by defects, of vinyl are largest ($\simeq$ 3.0 eV) because its C=C bond is at the side of the chain.

The C=O bond in C=O and HO–C=O defects includes one *π*_CO_ bond, so the extra electron trapping levels are determined by the energy levels of the ${\pi }_{{\rm{CO}}}^{\ast }$ orbital. In the cases of OH, C–O–C_ring_, and O–C–O defects, the only ${\sigma }_{{\rm{CO}}}^{\ast }$ orbital is higher in energy than the ${\sigma }_{{\rm{CH}}}^{\ast }$ orbital due to the same reason. Thus, no or very shallow electron trapping levels were observed in these cases, as can be seen in Fig. 1(b). Unlike unsaturated bond defects, the additional hole trapping levels induced by oxygen-containing groups are determined by the energy levels of the two lone-pair electrons of O, i.e., non-bonding orbitals. The deviations of the hole trapping levels of these defects may be attributed to the difference in their O environment. For instance, the O atom of the C–O–C_ring_ defect is located at the side chain while for O–C–O defect, O is a part of the backbone.

C–halogen bonds are highly polarizable, and the ${\sigma }_{{\rm{C}}-{\rm{halogen}}}^{\ast }$ orbital is lower in energy than the ${\sigma }_{{\rm{CH}}}^{\ast }$ orbital, leading to additional electron trapping levels. Because the large radius of the halogens reduces the C–halogen orbital overlaps, the electron trapping level of C–I bonds is lowest. Similar to O-containing groups, hole trapping levels of C–halogen systems are governed by the non-bonding orbitals of halogens. The computed ${E}_{{\rm{t}}}^{{\rm{e}}}$ of the C–Cl defect is low, presumably because of its strong polarization.

The computed thermodynamic and optical charge transition levels, e.g., ${E}_{(0/\pm 1)}^{{\rm{therm}}}$ and ${E}_{(0\leftrightarrows \pm \mathrm{1)}}^{{\rm{opt}}}$, are shown in Fig. 1(b). The differences between ${E}_{\mathrm{(0/}\pm \mathrm{1)}}^{{\rm{therm}}}$ and ${E}_{\mathrm{(0}\leftrightarrows \pm \mathrm{1)}}^{{\rm{opt}}}$ are due to the structural relaxation of PE in the vicinity of the defects during charging and discharging which are included in ${E}_{\mathrm{(0/}\pm \mathrm{1)}}^{{\rm{therm}}}$ but not in ${E}_{\mathrm{(0}\leftrightarrows \pm \mathrm{1)}}^{{\rm{opt}}}$. Because ${E}_{\mathrm{(0/}\pm \mathrm{1)}}^{{\rm{therm}}}$ and ${E}_{\mathrm{(0}\leftrightarrows \pm \mathrm{1)}}^{{\rm{opt}}}$ were computed from the total energy, they are physically relevant and thus, can be used to unveil the origins of electro-, photo-, and thermo-luminescences^3, 37^ as well as other experimentally measured properties of PE.

Indeed, the measured transport activation energy *E*_a_ may be associated with energy differences between the band edges and the charge transition levels. By placing the CBM and VBM with respect to ${E}_{\mathrm{(0/}\pm \mathrm{1)}}^{{\rm{therm}}}$ and ${E}_{(\pm 1\leftrightarrows \mathrm{0)}}^{{\rm{opt}}}$, the electron and hole activation energies, i.e., ${E}_{{\rm{a}}}^{{\rm{e}}}$ and ${E}_{{\rm{a}}}^{{\rm{h}}}$, were computed and summarized in Table 1. Computed ${E}_{{\rm{a}}}^{{\rm{e}}}$ of C=O, HO–C=O and O–C–O agree well with those obtained by X-ray thermally stimulated current experiments (1.4 eV) for oxidation^28^, indicating that C=O and O–C–O may serve as deep electron traps in PE. This claim may also be supported by ${E}_{{\rm{a}}}\simeq 2.0\,{\rm{eV}}$ extracted from mobility measurements of oxidized high-density PE (HDPE)^49^. For PE with C–I bonds, the upper limit of ${E}_{{\rm{a}}}^{{\rm{e}}}\simeq 0.96\,{\rm{eV}}$ explains well the origin of measured *E*_a_ of 0.85 eV for the electronic conduction^50, 51^.

**Table 1:** Computed trap depths (${E}_{{\rm{t}}}^{{\rm{e}}}$ and ${E}_{{\rm{t}}}^{{\rm{h}}}$) and electron and hole activation energies (${E}_{{\rm{a}}}^{{\rm{e}}}$ and ${E}_{{\rm{a}}}^{{\rm{h}}}$), given in eV, of PE with chemical defects.

Defects	${{\boldsymbol{E}}}_{{\bf{t}}}^{{\bf{e}}}$	${{\boldsymbol{E}}}_{{\bf{t}}}^{{\bf{h}}}$	${{\boldsymbol{E}}}_{{\bf{a}}}^{{\bf{e}}}$	${{\boldsymbol{E}}}_{{\bf{a}}}^{{\bf{h}}}$	Expt.
C=C	0.64	1.38	1.00–1.46	0.65–0.84	—
C=C–C=C	1.68	1.94	1.86–2.06	1.13–1.36	
C=CH_2_	2.77	3.36	1.69–1.81	2.96–3.10	
C=O	1.46	0.83	1.30–2.17	0.34–0.40	1.40^28^2.0^49, 50^
OH	0.00	0.43	0.20–0.21	0.96–0.97	
HO–C=O	1.02	0.51	0.38–1.36	1.93–2.27	
C–O–C_ring_	0.00	1.07	0.11–0.30	1.06–1.13	
O–C–O	0.25	0.67	0.96–2.23	0.52–0.57	
C–I	1.09	1.54	0.05–0.96	2.60–2.70	0.85^50, 51^
C–Br	0.64	0.87	0.60–1.84	1.59–1.67	—
C–Cl	0.14	0.41	0.55–2.20	1.23–1.28	—

Experimental values are given when available.

#### Physical disorders

Because crystalline regions of PE are generated by cooling the molten states, physical imperfections, including density variations, branches, cross links between chains, non-uniform bond length and angles (referred to as NU-*l*_AB_/∠_ABC_, where A, B, C are atoms), and some conformational disorders (e.g., gauche conformations (conf.) and kinks), are inevitably present in PE^41, 42^, as shown in Fig. 2(c). The electronic band diagrams computed for PE with these disorders are shown in Fig. 1(b), while the computed trap depths, i.e., ${E}_{{\rm{t}}}^{{\rm{e}}}$ and ${E}_{{\rm{t}}}^{{\rm{h}}}$, are also given in Table 2.

**Table 2:** Computed electron and hole trap depths (${E}_{{\rm{t}}}^{{\rm{e}}}$ and ${E}_{{\rm{t}}}^{{\rm{h}}}$), given in eV, of PE with physical disorders.

Configurations			*ρ*	${{\boldsymbol{E}}}_{{\bf{t}}}^{{\bf{e}}}$	${{\boldsymbol{E}}}_{{\bf{t}}}^{{\bf{h}}}$	Expt.
Perfect PE			1.08	0.00	0.00	—
Crystal region	Density variation		1.03	0.19	0.00	1.2–1.4^†^ (LDPE); 1.7^†^ (HDPE)
			0.98	0.33	0.00	
			0.90	0.53	0.00	
			0.83	0.71	0.00	
			0.77	0.84	0.00	
			0.72	0.94	0.00	
			0.54	1.29	0.00	
			0.36	1.34	0.00	
	Branches	CH_3_	0.56	1.24	0.09	0.24^‡^
		C_2_H_5_	0.57	1.21	0.12	
		C_3_H_7_	0.58	1.23	0.12	
		C_4_H_9_	0.60	1.25	0.10	
	Cross links	C_5_H_8_	0.61	1.11	0.27	
	NU-*l*_AB_/∠_ABC_		1.08	0.07	0.29	0.32–0.35^†‡^
	Gauche conf.		1.08	0.09	0.96	
	Kinks		0.84	0.77	0.81	1.2^‡^
Large-scale disorders	Lamella		0.99	0.88	1.07	—
	Amorphous		0.94	0.84	0.87	0.8–1.0^†^
	Semi-crystalline		0.97	0.93	1.09	1.0–1.4^†^; 1.2^‡^; 0.92^‡^

Experimental values of ${E}_{{\rm{t}}}^{{\rm{e}}}$ and ${E}_{{\rm{t}}}^{{\rm{h}}}$ are given when available. The density *ρ* of PE (with disorders) is given in g/cm^3^.

^†^Ref. 28; ^‡^Refs 49, 50 and 55; ^†‡^Ref. 54.

Figure 1(b) shows that when the density *ρ* varies from 0.36 g/cm^3^ to 1.03 g/cm^3^, the VBM is essentially the same with that of perfect PE while the CBM is dramatically shifted down. This implies that electrons prefer to transfer from high- to low-*ρ* regions, as previously indicated^52^. The reason is that in low-*ρ* PE, the large inter-chain distance reduces the anti-bonding hybridization, lowering the conduction state energies. When this distance is large enough (corresponding to $\rho \simeq 0.54\,{\rm{g}}/{{\rm{cm}}}^{3}$), ${E}_{{\rm{t}}}^{{\rm{e}}}$ saturates at $\simeq$ 1.3 eV. This value, which is consistent with ${E}_{{\rm{t}}}^{{\rm{e}}}\simeq 1.2-1.4$ measured for low-density PE (LDPE) and 1.7 eV measured for HDPE^28^, can occur at micro-voids, voids, and cavities of low-*ρ* PE.

Branches and cross links typically expand the host crystal lattices^53^, thus they were constructed in low-*ρ* PE supercells (*ρ* = 0.54 g/cm^3^). With these disorders, *ρ* is slightly raised, moving the CBM up. For branches, the VBM shift of ${E}_{{\rm{t}}}^{{\rm{h}}}\simeq 0.1\,{\rm{eV}}$, originating from the replacement of a *σ*_CH_ bond by a *σ*_CC_ bond, depends weakly on the length of the branches. In the case of cross links, such replacement occurs at both ends of the linking chains, and a deeper ${E}_{{\rm{t}}}^{{\rm{h}}}\simeq 0.27\,{\rm{eV}}$ is observed. This reveals that the measured *E*_a_ of 0.24 eV^50^ from transient current is derived from hole-transport induced by branches.

NU-*l*_AB_/∠_ABC_ can reduce the orbital overlaps, ultimately modifying the valence band edges. Bond length elongation diminishes the *σ* bonding, giving rise to a shallow ${E}_{{\rm{t}}}^{{\rm{h}}}\simeq 0.29\,{\rm{eV}}$, being consistent with the trap depth of $\simeq \,0.32\,\mbox{--}\,0.35{\rm{e}}{\rm{V}}$ measured for LDPE and HDPE^54^. For gauche conformations, changes in the C–C–C–C torsion angles can dramatically reduce their orbital overlaps, introducing an ${E}_{{\rm{t}}}^{{\rm{h}}}$ of up to 0.96 eV. An analysis of the projected density of states of kinks, composed of 1 gauche + *n* all-trans + 1 gauche conformations, reveals that their VBM is dominated by the gauche part, unraveling the similarity in VBM between kinks and gauche conformations. The resulting ${E}_{{\rm{t}}}^{{\rm{h}}}\simeq 1.0\,{\rm{eV}}$, which is consistent with the measured *E*_a_ of 1.2 eV for twisting chains^55^, suggests that the hole-transport process can be enhanced by the presence of gauche conformations and kinks.

The electronic band diagrams of PE with large-scale disorders, i.e., lamella, amorphous, and semi-crystalline, are portrayed in Fig. 1(b). Similar to gauche conformations and kinks, the VBM of lamella and semi-crystalline PE are attributed to the C–C–C–C torsion angles of $\simeq$ 60° in the folding segments of the PE chains while low-*ρ* regions are responsible for the drop of the CBM. In semi-crystalline PE, the density of the lamellas/amorphous regions interfaces is very low, thus the CBM is further lowered. We suggest that the low-*ρ* interface regions play an important role in the conduction of PE because electrons prefer to accumulate here^52^. Different from previous calculations^39^, our computed ${E}_{{\rm{t}}}^{{\rm{e}}}$ of amorphous and lamella/amorphous interface disordered PE are close to the measured trap depths^28^. Moreover, ${E}_{{\rm{t}}}^{{\rm{e}}}$ computed for semi-crystalline PE agrees well with that experimentally obtained from transient space charge limited current peak and surface charge decay of HDPE (1.2 eV) and LDPE (0.92 eV)^49, 50^.

#### Implications

Some remarks can be made based on Tables 1 and 2. First, OH and C–O–C_ring_ chemical defects, local densities of $\mathop{ < }\limits_{ \tilde {}}$ 1.03 g/cm^3^, cross links, branches, and NU-*l*_AB_/∠_ABC_ can lead to shallow ${E}_{{\rm{t}}}^{{\rm{e}}}$ and/or ${E}_{{\rm{t}}}^{{\rm{h}}}$ (< 0.3 eV)^33^. Second, amorphous, lamella/amorphous interfaces, and chemical defects (except OH and C–O–C_ring_) can cause deep electron traps, assisting electron transport between traps and the conduction bands, i.e., trap-controlled band conduction, and enhancing electron conduction. Supporting evidence includes the high electron mobility experimentally observed via surface charge decay^28, 50^. Third, kinks, gauche conformations, folded PE chains, and chemical defects can introduce deep hole traps, enhancing the hole transport. Unfortunately, experimental evidence remains unclear due to technical challenges. Finally, hole trapping levels of kinks and lamella are close to those of C=C, OH, C=O, and C–O–C_ring_. Similar observations can also be found for the electron trapping levels of some physical disorders and chemical defects, suggesting tunneling or thermally activated hopping transport. As an example, a conduction electron can transfer from one PE chain into a low-*ρ* region, then being injected into a chemical defect of another chain. Likewise, a hole can be formed when an electron is injected into kinks, then it can transfer along the PE chain before being captured by hole traps induced by chemical defects in other chains. This may be a reason for the high mobility of oxidized PE and I_2_ doped PE^50^.

### Electrode/Polyethylene interfaces

Charge injection, occurring at the interface between a PE slab and a metal electrode, is the initiating factor leading to the degradation of this polymer. Thus, understanding the electronic structure of such interfaces is vital. Herein, we present a comprehensive study of Al/PE interfaces, constructed and shown in Fig. 2(d), as a prototype. We assumed that such interfaces were fabricated by depositing an Al layer on an oxygen-treated PE film^46, 56^, inevitably forming some O-containing groups close to the interface.

#### Interface structures

In the “clean” Al/PE interface, i.e., that without O-containing defects, PE and Al slabs are separated by $\simeq$ 3.1 Å, indicating that only physical interactions exist between the PE and the Al slabs. Due to the large electronegativity of O, the interaction between the Al and the PE+O–C–O slabs becomes stronger, evidenced by a distance of $\simeq$ 2.6 Å between the slabs. For the Al/PE+OH interface, surface Al atoms move toward the PE slab, forming metastable Al–O bonds of $\simeq$ 2.1 Å in length. As shown in Fig. 2(d), such Al–O bonds are formed either by breaking a C=O double bond (in the Al/PE+C=O or Al/PE+HO–C=O interfaces), or a C–O bond (in the Al/PE+C–O–C interface). Al–C bonds (of $\simeq$ 1.8–2.0 Å in length) were also observed in the Al/PE+C–O–C_ring_ and Al/PE+C=O interfaces. Consequently, the work of separation *W* (computed as $W={E}_{{\rm{Al}}/{\rm{PE}}}-{E}_{{\rm{Al}}}-{E}_{{\rm{PE}}}$ from the DFT energies of Al/PE interface, pure Al slab, and PE slab) for these two interfaces is much larger than that of the clean Al/PE interface, as shown in Table 3. More importantly, these strongly polarized bonds may greatly impact the interfacial dipole moments and hence, the charge injection barriers^40^.

**Table 3:** Computed work of separation (*W*, in J/m^2^), vacuum level shift (Δ*φ*, in eV) and electron and hole injection barriers (*ϕ*_e_ and *ϕ*_h_, in eV) of Al/PE interfaces (with and without imperfections).

Interface region			Bulk region of PE					
Configurations	*W*	Δ*φ*	Perfect crystal		Physical disorders		Chemical defects	
			*ϕ*_e_	*ϕ*_h_	*ϕ*_e_	*ϕ*_h_	*ϕ*_e_	*ϕ*_h_
Clean Al/PE	0.37	−0.20 (−0.30^57, 61^)	4.11	4.17	2.77–4.04	3.08–4.17	1.34–4.11	0.81–3.66
Al/PE+O–C–O	0.71	−1.03 ± 0.53	3.28	5.00	1.94–3.21	3.91–5.00	0.51–3.32	1.63–4.49
Al/PE+OH	0.87	−1.38 ± 0.73	2.93	5.35	1.59–2.86	4.26–5.35	0.16–2.96	1.99–4.84
Al/PE+HO–C=O	0.91	−1.50 ± 0.27	2.81	5.47	1.47–2.73	4.38–5.47	0.03–2.84	2.11–4.97
Al/PE+C–O–C_ring_	1.76	−1.56 ± 1.07	2.75	5.53	1.41–2.68	4.44–5.53	0.00–2.79	2.16–5.02
Al/PE+C=O	1.82	−1.62 ± 0.70	2.69	5.59	1.35–2.62	4.50–5.59	0.00–2.73	2.22–5.08

Experimental data, when available, is given in brackets.

#### Interface vacuum energy shift

**Figure 3:**
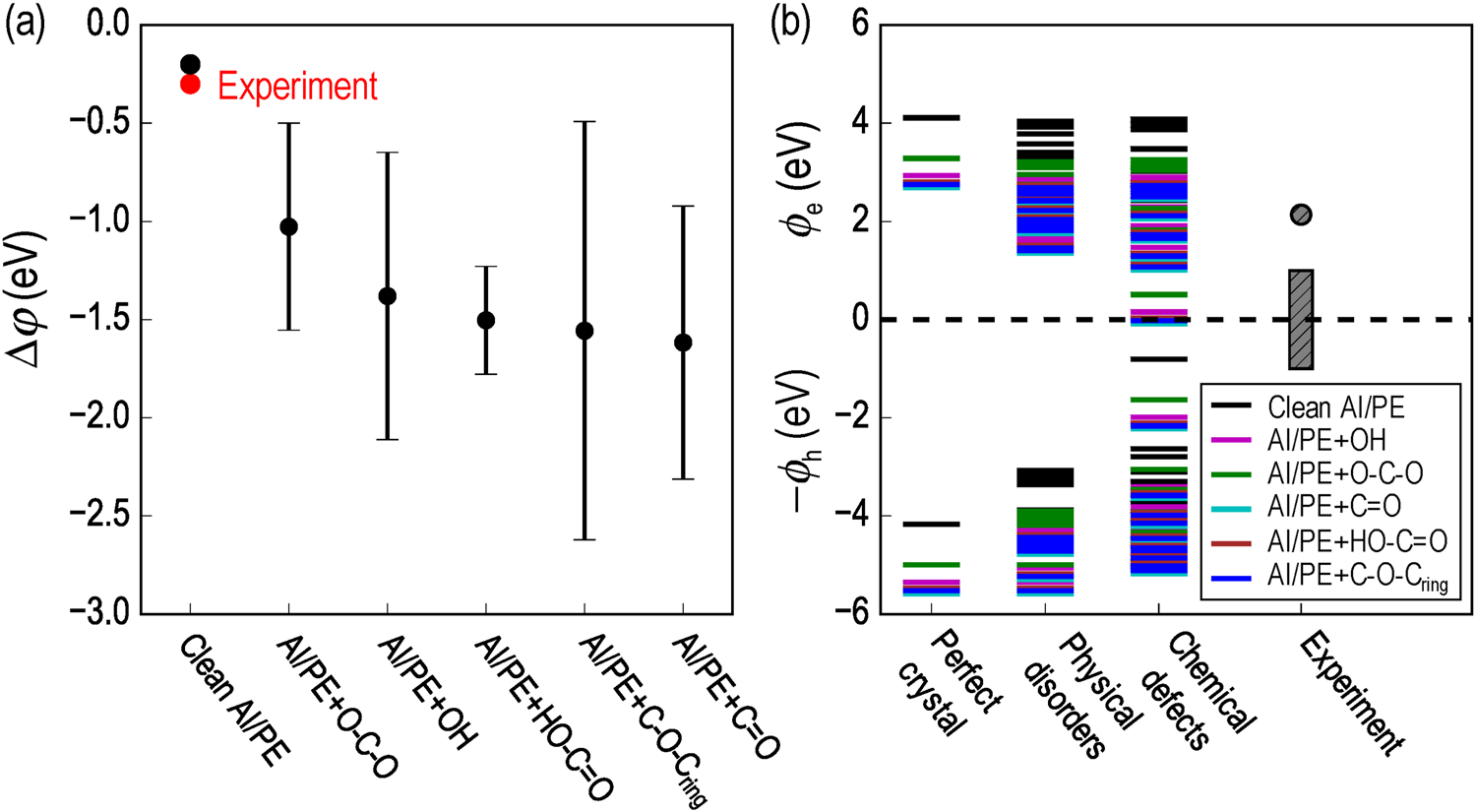
(**a**) Computed vacuum level shift Δ*φ* at Al/PE interfaces, of which the error bars are obtained from the standard deviations of t﻿he results from different orientations of the O-containing groups. (**b**) Electron and hole injection barrier *ϕ*_e_ and *ϕ*_h_ computed for the Al/PE interface, of which the PE slab may or may not contain physical disorders and/or chemical defects. Experimental values of the barriers are taken from Refs 33 and 62.

Because of the interfacial dipole moment **D**, the vacuum energy levels at Al and PE sides are misaligned by Δ*φ*, as illustrated in Fig. 1(b). Computed values of Δ*φ* are shown in Table 3 and Fig. 3(a). For the interfaces considered, Δ*φ* is negative, signaling a downward shift of the vacuum energy level in the PE side. The increasing trend of Δ*φ* from Al/PE to Al/PE+O–C–O to Al/PE+OH to Al/PE+HO–C=O and to Al/PE+C=O indicates the growing strength of **D**. This dipole moment originates from the rearrangement of the electron density distribution across the two sides of the interface, driven by the Pauli repulsion^57–59^. Such a “pillow effect”^60^ drops the PE vacuum level by $\simeq$ 0.2 eV, consistent with the downward shift of $\simeq$ 0.3 eV measured for a *n*-CH_3_(CH_2_)_44_CH_3_ (tetratetracontane) monolayer absorbed on an Al(111) surface^57, 61^, of which the structure is similar to the Al/PE interface examined.

The permanent dipole moments induced by the O-containing groups depend strongly on their orientation. Thus, three positions of such groups in the second layer of the interface region were considered, resulting in a range of calculated Δ*φ* shown in Fig. 3(a). The most/less negative value of Δ*φ* was obtained with parallel/anti-parallel O-containing groups in the top two layers. For Al/PE+C–O–C_ring_ interface, Δ*φ* can be as large as −2.62 eV while for Al/PE, Δ*φ* is rather small when the O-containing groups are anti-parallel. Computed Δ*φ* of Al/PE+O–C–O is larger ($\simeq$ −0.5 eV) due to the electronegativity of O while for Al/PE+OH, Al/PE+HO–C=O, Al/PE+C–O–C_ring_ and Al/PE+C=O interfaces, Δ*φ* is more negative. The reason is that in addition to the permanent dipole moments, Al–O and Al–C bonds also contribute to **D** by rearranging the interface charges.

In summary, the primary factors that are responsible for **D** and Δ*φ* are the “pillow effect”, the permanent dipole of polar groups, and the formation of polar bonds, of which the last two factors are dominant. Both of them may be introduced by the O-containing groups, significantly dropping the vacuum energy level in the PE side (by $\mathop{ > }\limits_{ \tilde {}}$ 1.0 eV) and greatly affecting the charge injection barriers.

#### Charge injection barriers

Given the Δ*φ* determined, the electron and hole injection barriers of the Al/PE interfaces, i.e., *ϕ*_e_ and *ϕ*_h_, defined as the energy difference between the Fermi level and the PE band edges or trap levels (see Fig. 1(b)), were computed and summarized in Table 3 and Fig. 3(b). By definition, these barriers are important characteristics of the interface, governing the charge injection from the Al electrode into PE. Without imperfections, the computed barriers ($\simeq$ 4.0 eV) are too high compared to the experimental values ($\simeq$ 1.00 – 2.14 eV)^33, 62^. We found that the imperfections considered (chemical defects, physical disorders, interfacial polar groups, and the formation of the polar bonds) strongly alter the electronic structure of PE, lowering *ϕ*_e_ and *ϕ*_h_ to $\simeq$ 0.0–2.0 eV, and covering the whole range of the experimental data.

## Discussion and Summary

The electronic structure of realistic models of an insulator, which is currently accessible via computations, is a key gateway towards understanding the electrical degradation phenomena. Because realistic polymers comprise of exceedingly complicated interface morphologies and multi-scale chemical defects and physical disorders, properly modeling them in a consistent level of theory is challenging and has not been previously performed thoroughly.

We have presented a comprehensive picture of the electronic structure of realistic PE, systematically examining a majority of inevitable imperfections in this polymer, including chemical, physical, interfacial, and morphological defects and disorders. By constructing enormous models (some of which contain up to 2,400 atoms) and properly combining the beyond-conventional DFT with molecular dynamics simulations, the proposed computational approach has reached an excellent level of accuracy in determining defect levels, activation energies, trap depths of PE, and the charge injection barriers at the interface between PE and Al electrodes. The obtained electronic structure provides a basis to better understand the existing experimental data involving the luminescence characteristics, the high field conduction, and thus, the long-term degradation of PE. The reported results, e.g., those given in Fig. 1(b), can be input variables for building phenomenological transport models in which the densities and trap depths of various defects and charge injection barriers are required.

Overall, the key findings of this work, which include not only the numerical results but also the insights into relevant physical and chemical processes, could take us a step closer to the control of polymer degradation and the rational design of breakdown-resistant polymer dielectrics. The computational scheme described herein is reliable, generalizable, and thus being applicable to realistic studies of any insulators.

## Methods and Materials

### General computational scheme

First-principles calculations were performed using the density functional theory (DFT) method^47, 48^ as implemented in the Vienna *ab*-*initio* simulation package (vasp)^63^. Monkhorst-Pack **k**-point meshes^64^ used for our calculations are summarized in Table 4. Except kinks and large-scale physical disorders, for which the structures were obtained directly from MD simulations, the structures with other defects/disorders were relaxed using the Perdew-Burke-Ernzerhof (PBE) XC functional^65^. The Tkatchenko-Scheffler functional was used for van der Waals interactions^66^. In calculations involving charged defects, first-order monopole corrections were used to correctly describe the electrostatic interactions of charged defects due to the periodicity and the finite supercell sizes. *Ab*-*initio* MD simulations were performed with vasp, while classical MD simulations were carried out with the reactive force field (ReaxFF)^67–69^, using the LAMMPS simulation package^70^. A time-step of 0.5 fs was used in all the MD simulations, for which involving NPT dynamics the simulation time was determined to obtain the convergence of densities at each specific temperature and pressure.

**Table 4:** Primary parameters, including the number of atoms *N*_at_ and the *k*-point meshes, used for our calculations.

Systems	*N*_at_	*k*-point
PE unit cell	12	4 × 4 × 10
Physical disorders (crystal region)	120	4 × 2 × 2
Large-scale physical disorders	1,202*/2,402**	1 × 1 × 1
Chemical defects	68–75	4 × 2 × 2
Al/PE interfaces	296–324	2 × 2 × 1

*Lamella/amorphous region of PE; **Semi-crystalline PE.

### Electronic structure calculations

Electronic structure calculations involving small imperfections but large-scale disorders were performed using the HSE06 XC functional^71^, believed^3, 30^ to be adequate for polymers, including PE. To extract the electron and hole trap depths, i.e., ${E}_{{\rm{t}}}^{{\rm{e}}}$ and ${E}_{{\rm{t}}}^{{\rm{h}}}$, Kohn-Sham eigenvalues were corrected by aligning the average C-1*s* core level state in the defect-containing models with those of perfect PE. For large-scale disorders, e.g., lamella, amorphous, and semi-crystalline, the hole trap depth ${E}_{{\rm{t}}}^{{\rm{h}}}$ was computed using the PBE XC functional, and the electron trap depth is obtained by ${E}_{{\rm{g}}}^{{\rm{HSE}}06}+{E}_{{\rm{t}}}^{{\rm{h}}}$, where ${E}_{{\rm{g}}}^{{\rm{HSE}}06}$ is the band gap estimated at the HSE06 level of DFT using Eq. (1). This relation was derived from ${E}_{{\rm{g}}}^{{\rm{PBE}}}$, the band gap calculated at the PBE level of DFT, and ${E}_{{\rm{g}}}^{{\rm{HSE}}06}$, both of them were computed for physical disorders in the crystal region (raw data is given in Fig. S2 of SI).

1 $${E}_{{\rm{g}}}^{{\rm{HSE}}06}=1.1028\times {E}_{{\rm{g}}}^{{\rm{PBE}}}+0.689,{R}^{2}=0.99$$

### Thermodynamic and optical charge transition levels

Thermodynamic transition levels (${E}_{(q/q^{\prime} )}^{{\rm{therm}}}$) is defined as the Fermi energy at which defects in charge states *q* and *q*′ are at thermodynamic equilibrium. It is given by Ref. 37

2 $${E}_{(q/q^{\prime} )}^{{\rm{therm}}}=\frac{{E}_{q}^{{\rm{f}}}({R}_{q})-{E}_{q^{\prime} }^{{\rm{f}}}({R}_{q^{\prime} })}{q-q^{\prime} }.$$

Here, ${E}_{q}^{{\rm{f}}}({R}_{q})$ is the formation energy of the *q*-charged defect at its equilibrium structure *R*_*q*_, which can be obtained from DFT calculations. The Fermi energy is taken from the VBM to the CBM of the defect-free PE. On the other hand, optical transition level corresponds to the charge transition of the defect, given that the atomic configuration is frozen. It is given as

3 $${E}_{(q\to q^{\prime} )}^{{\rm{opt}}}=\frac{{E}_{q^{\prime} }^{{\rm{f}}}({R}_{q})-{E}_{q}^{{\rm{f}}}({R}_{q})}{(q^{\prime} -q)}.$$

### Charge injection barriers computation

According to the energy-diagram shown in Fig. 1(b), due to the formation of interfacial dipole moments **D**, the PE and Al vacuum levels are misaligned by Δ*φ*, defined as ${\rm{\Delta }}\phi =-\frac{e{\bf{D}}}{{\varepsilon }_{0}A}$ ^40^. Here, ${\varepsilon }_{0}$ is the vacuum permittivity, *e* the electron charge, and *A* the area of Al/PE interface. **D** was computed by integrating the elementary dipole moment, obtained from DFT, over the whole system. The electron and hole injection barriers, i.e., *ϕ*_e_ and *ϕ*_h_, are given by

4 $${\varphi }_{{\rm{e}}}={\psi }_{{\rm{m}}}+{\rm{\Delta }}\phi -{E}_{{\rm{ea}}}-{E}_{{\rm{t}}}^{{\rm{e}}},$$

5 $${\varphi }_{{\rm{h}}}={E}_{{\rm{g}}}-{E}_{{\rm{t}}}^{{\rm{e}}}-{\varphi }_{{\rm{e}}}-{E}_{{\rm{t}}}^{{\rm{h}}}.$$

Here, ${\psi }_{{\rm{m}}}$, *E*_g_, *E*_ea_, ${E}_{{\rm{t}}}^{{\rm{e}}}$, and ${E}_{{\rm{t}}}^{{\rm{h}}}$ are the computed Al work function (4.26 eV), the computed band gap of perfect PE (8.28 eV), the electron affinity of the PE (110) slab (−0.05 eV), the electron and hole trap depths, respectively. Herein, ${\psi }_{{\rm{m}}}$ and *E*_ea_ were computed for individual Al (111) and PE (110) slabs using the DFT-based approach called “bulk plus band lineup” method^72^, being consistent with experimental values^40^.

### Physical disorder generation

Physical disorders were modeled in compliance with existing experimental data^41, 42^. Because such imperfections are generally large in scale, they were generated with system size ranging from 120 to 2,402 atoms. Within a 1 × 2 × 5 supercell (120 atoms) of crystalline PE, some (smallest) disorders were constructed. Density variation was captured by changing the inter-chain distance to obtain a density *ρ* range of 1.03–0.36 g/cm^3^ (for perfect crystalline PE, *ρ* = 1.08 g/cm^3^). Branches were created by replacing a hydrogen with a C_*n*_H_2*n*+1_ group (*n* = 1–4) while C_5_H_8_ chains were used to link PE chains, forming cross links disorders. NU-*l*_AB_/∠_ABC_ and gauche conformations were generated via first-principles NVT-MD simulations (*T* = 300 & 700 K, respectively) over 1 ps.

For kinks and larger disorders, MD simulations with ReaxFF was used. Kinks were generated using NPT-ensemble MD (*P* = 1 atm & *T* = 520 K) over 100 ps, for which the parameters were determined from the phase diagram in Fig. S4 of SI. A lamella was generated using a multi-step procedure. First, an NVT-MD simulation at *T* = 300 K was performed over 200 ps, starting from a supercell containing a folded PE chain of 1,202 atoms. A NPT-MD simulation (*P* = 1 atm & *T* = 300 K) followed, resulting in a reasonable density. Amorphous disorders were generated by simulating the lamella configurations with NVT MD at *T* = 600 K over 100 ps. The temperature of the obtained liquid PE was then lowered to 300 K during the second NVT-MD simulation over 100 ps before the last MD simulation with NPT ensemble (*T* = 300 K) is carried out for 200 ps. The preparation of semi-crystalline PE structures included two MD simulations performed on a supercell of 2,402 atoms, prepared by combining the lamella and amorphous equilibrium structures. First, an NVT (*T* = 300 K) MD simulation was performed during 10 ps, and then, an NPT (*P* = 1 atm & *T* = 300 K) simulation followed for 400 ps. The resulting density of 0.97 g/cm^3^ is compatible with that of HDPE^41^. Except branches and cross-link disorders, 10 configurations were either generated separately (with different *a*/*b* ratio) for density variation or randomly selected from the equilibrated MD trajectories for other disorders.

### Al/PE interface constructions

Starting from an ideal (absolutely flat) interface between a PE and an Al (111) slab, two *ab initio* MD simulations were consecutively performed at *T* = 300 K and 600 K for 1 ps. During the MD runs, only the interface region (see Fig. 2(d)) was relaxed while the other regions were fixed. The whole Al/PE structure were then optimized using DFT (at 0 K). The close-packed (111) plane of Al was selected for minimizing the lattice mismatch between the Al and crystalline PE slabs. Because the computed work functions of different Al planes are similar, we expect that the Al (111) plane is a good representative to study the charge injection barrier at Al/PE interfaces^73^.

## Electronic supplementary material

Supplementary Information
