# Supplementary material for: Electronic Structure of Polyethylene: Role of Chemical, Morphological and Interfacial Complexity
Source: Sci Rep. 2017 Jul 21;7:6128. doi: 10.1038/s41598-017-06357-y (PMC5522474; doi:10.1038/s41598-017-06357-y)
Supplement: Supplementary file 1 — Supplementary Information [file 41598_2017_6357_MOESM1_ESM.pdf]

Supplementary Information

Electronic Structure of Polyethylene: Role of Chemical, Morphological and Interfacial Complexity

Lihua Chen, Tran Doan Huan, and Rampi Ramprasad*

Department of Materials Science & Engineering and Institute of Materials Science, University of Connecticut, 97 North Eagleville Rd., Unit 3136, Storrs, CT 06269-3136, USA

Supplementary Information Available:

1. Computed electronic structure of crystal PE (Fig. S1)
2. Relationship between $E_{\mathrm{g}}^{\mathrm{PBE}}$ and $E_{\mathrm{g}}^{\mathrm{HSE06}}$ (Fig. S2)
3. Validation of reactive force field (ReaxFF):
   Geometry parameters of crystal PE (Table S1);
   Thermal density variations of crystal PE (Fig. S3);
   Phase diagram of PE (Fig. S4)

*Corresponding author; email: rampi.ramprasad@uconn.edu

1 Computed electronic structure of crystal PE

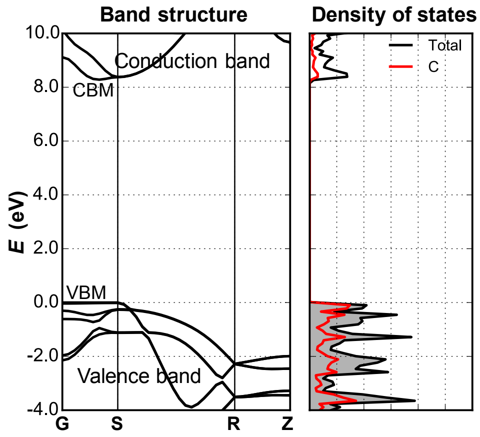

Fig. S1: HSE06 electronic structure of the perfect crystal PE whose VBM is set to 0 eV, along with density of states.

2 Relationship between E_g^{PBE} and E_g^{HSE06}

The band gap of physical disorders in the crystal region were computed both with the PBE and HSE06 functionals, i.e., E_g^{PBE} and E_g^{HSE06}. 50 data was selected to cover a wide range of the band gap, including 1 from the perfect crystal, 19 from the density variation, 10 from the non-uniform bonds/angles, 10 from the gauche conformations, and 10 from the kinks. It can be seen from Fig. S2, there is a linear relationship between E_g^{PBE} and E_g^{HSE06}, i.e., $E_g^{HSE06} = 1.1028 \times E_g^{PBE} + 0.689, R^2 = 0.99$. All original data is available in $http://khazana.uconn.edu$.

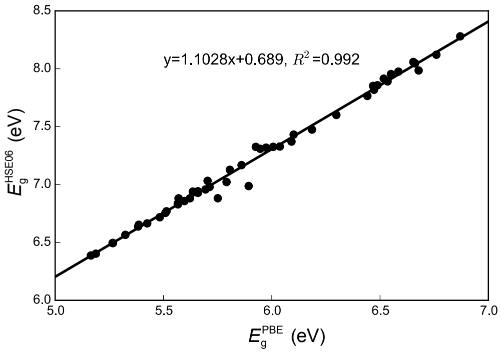

Fig. S2: Relationship between E_g^{PBE} and E_g^{HSE06}, derived from calculations involving physical disorders in the crystal region.

3 Validation of reactive force field (ReaxFF)

3.1 Geometry parameters of crystal PE

Table S1 lists geometric parameters of crystal PE relaxed with the PBE functional and MD simulations with ReaxFF at 0 K, along with experimental values [1]. Moreover, relaxed bond lengths (l, in Å) and angles ($\angle$, in deg.) of a 1×2×5 supercell of PE are also included, obtained from ab-initio and ReaxFF based MD simulations at NVT ensemble (T = 300K). Comparing with experimental and PBE results, it can be concluded that ReaxFF provides an acceptable accuracy to model PE.

Table 1: Geometric parameters of crystal PE relaxed with the PBE functional and ReaxFF-MD simulations at 0 K, together with experimental results from Ref. [1]. Variations of bond lengths (l) and angles ($\angle$) of a 1×2×5 supercell of PE, computed with ab-initio MD with the PBE functional and ReaxFF-MD at NVT ensemble with T = 300 K.

Geometric parameters	0K		300K		Expt.
	PBE	ReaxFF-MD	ab-initio MD	ReaxFF-MD	
a (Å)	6.962	6.982			7.42±0.01
b (Å)	4.822	4.812			4.95±0.01
c (Å)	2.560	2.559			2.55±0.004
l_{C-C} (Å)	1.528	1.558	1.557±0.048	1.660±0.104	1.530
l_{C-H} (Å)	1.102	1.117	1.122±0.053	1.120±0.046	1.090
$\angle_{C-C-C}$ (deg.)	113.77	110.407±0.001	114.468±6.027	110.86±1.759	112±0.3
$\angle_{C-C-H}$ (deg.)	109.36	110.598±0.020	109.263±5.978	110.44±3.079	109.5

3.2 Thermal density variations of crystal PE

With ReaxFF, a 2×2×10 supercell of PE structure was equilibrated by MD simulations at NPT ensemble over 1 ns, where T in a range of 150 − 700 K and pressure at 1 atm. The resulting densities at different T are shown in Fig. S3, within 10% of available experiments [2]. Moreover, it can be observed that the density decreases sharply at T = 520 K (dashed line), which is defined as the melting point T_m.

3.3 Phase diagram of PE

Following the same method described in Sec. 3.2, T_m of crystal PE at different pressure (i.e., 1, 250, 500, 780, 1000 atm) were predicted. These information was used to generate the phase diagram of PE, as shown in Fig. S4, in which T_m at different pressure becomes a boundary between solid and liquid phases. Experimental [3] and computed results are denoted by black and red lines, respectively. It can be seen that the deviation between predicted and experimental melting points is about 100 K, which is acceptable.

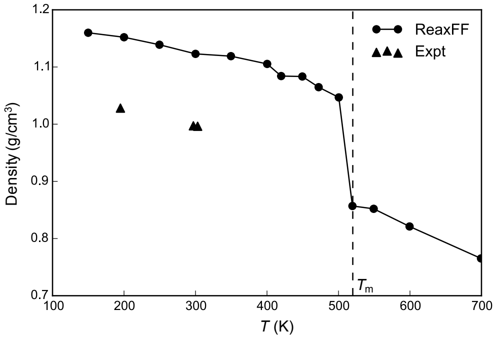

Fig. S3: Density (g/cm^3) of crystal PE at different temperature (T, in K), obtained from MD simulations with ReaxFF at pressure of 1 atm (circles) and experimental data from Ref. [2] (triangles). The dashed line corresponds to the melting point (T_{m}).

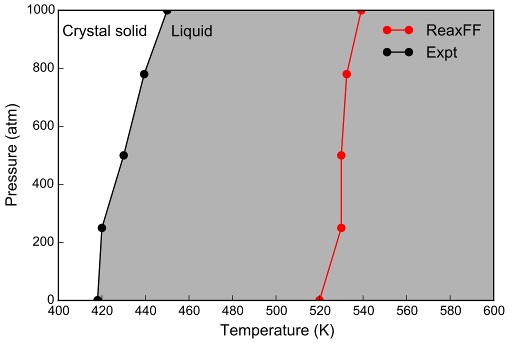

Fig. S4: Phase diagram of PE with solid and liquid phases denoted by blank and shaded regions, respectively. The red and black circle lines are phase boundaries obtained from MD simulations with ReaxFF and experimental values from Ref. [3], respectively.

References

[1] Montanari, B., & Jones, R. Density functional study of crystalline polyethylene. *Chem. Phys. Lett.* **272**(5), 347–352 (1997).

[2] Sun, H. Compass: an ab-initio force-field optimized for condensed-phase applications overview with details on alkane and benzene compounds. *J. Phys. Chem. B* **102**(38), 7338–7364 (1998).

[3] Androsch, R., Di Lorenzo, M. L., Schick, C., & Wunderlich, B. Mesophases in polyethylene, polypropylene, and poly (1-butene). *Polymer* **51**(21), 4639–4662 (2010).
